# Histopathological study and intestinal mucous cell responses against *Aeromonas hydrophila* in Nile tilapia administered with *Lactobacillus rhamnosus* GG

**DOI:** 10.14202/vetworld.2020.967-974

**Published:** 2020-05-22

**Authors:** Suchanit Ngamkala, Khomson Satchasataporn, Chanokchon Setthawongsin, Wuttinun Raksajit

**Affiliations:** Department of Veterinary Technology, Faculty of Veterinary Technology, Kasetsart University, Bangkok 10900, Thailand

**Keywords:** *Aeromonas hydrophila*, intestine, *Lactobacillus rhamnosus* GG, mucous cell, Nile tilapia

## Abstract

**Aim:**

This study aimed to examine the intestinal histopathological lesions and mucous cell responses in the entire intestines of Nile tilapia administered with *Lactobacillus rhamnosus* GG (LGG)-mixed feed, after *Aeromonas hydrophila* challenge.

**Materials and Methods:**

Intestinal samples from fish fed with control normal diet or LGG-mixed feed (10^10^ colony-forming unit [CFU]/g feed) with or without *A. hydrophila* in phosphate-buffered saline challenge (7.46 × 10^8^ CFU/mL/fish) were collected and processed for histopathological study. The mucous cell responses were evaluated using histochemistry, using Alcian blue (AB) at pH 2.5, AB at pH 1.0, and periodic acid-Schiff-AB at pH 2.5. The quantification of the intestinal mucous cell size and the staining character of each mucin type from the entire intestine were recorded and counted.

**Results:**

Histopathological study showed remarkable lesions only in the proximal intestine in fish infected with *A. hydrophila*, while LGG-fed fish had less intestinal damage, perhaps resulting from heterophil infiltration. Furthermore, a significant (p<0.01) increase in mixed mucous cell numbers was observed mainly in the proximal intestine of all challenged fish, compared with normal diet-fed fish without challenge, and also in LGG-fed fish with *A. hydrophila* challenge compared with LGG-fed fish without challenge.

**Conclusion:**

Dietary LGG-fed Nile tilapia showed improvements in host innate immunity. In addition, LGG was effective in decreasing intestinal lesions from *A. hydrophil*a-induced intestinal damage. Moreover, increasing numbers of mixed mucous cells in the proximal intestine might be indicative of certain pathological conditions in Nile tilapia after *A. hydrophila* infection.

## Introduction

Nile tilapia (*Oreochromis niloticus*) is a freshwater fish which is able to tolerate various conditions in both tropical and non-tropical areas. Farming of Nile tilapia is highly valuable to the Thai economy [1]. However, infectious diseases affecting Nile tilapia appear to be a major cause of economic losses. *Aeromonas* infection is among the most important bacterial diseases in fish; the severity of infection depends on the strain of pathogen. The most severe type of infection is caused by *Aeromonas hydrophila*, which is commonly found in freshwater [2,3]. *A. hydrophila* infection can also cause septicemia and immunosuppression, resulting in hemorrhage and skin ulceration, detached scales, ascites, skin darkness, and exophthalmos [4]. This disease is present in various types of fish [5]. Therefore, enhancing infectious disease prevention and control seems a promising method for improving production efficiency. Another key point for the improvement of fish health is that an efficient immune response can help to reduce clinical signs, pathological changes, morbidity, and mortality rates.

There have been previous studies on the use of various immunostimulants and their outcomes in fish [6,7]. Many studies have demonstrated that application of probiotics can promote both innate and adaptive immunity in fish [8,9]. Probiotics are live, beneficial microbes which can modulate and improve host immune function and disease resistance when allocated in appropriate quantities [10]. The application of probiotics in aquaculture is considered a good alternative to the use of antibiotics thanks to the former’s higher safety record and the beneficial reduction in the use of drugs and/or chemical substances. General considerations when selecting a probiotic are biosafety, production procedure, administration method, and expected target site for colonization and activation in the body [11,12]. In aquaculture, Gram-positive bacteria (*Micrococcus luteus*, *Streptococcus thermophilus*, *Bacillus* sp., *Carnobacterium* sp., and *Lactobacillus* sp.), Gram-negative bacteria (*Pseudomonas fluorescens*, and Vibrio sp.), yeasts (*Saccharomyces cerevisiae*), microalgae (*Tetraselmis suecica*), and bacteriophages have all previously been used to enhance the growth performance, digestive enzyme activity, and immunity [6,13]. Among the most commonly used probiotics are *Lactobacillus* sp., from the lactic acid bacteria (LAB) family. This bacterial family is the dominant bacteria in the human small intestine [14]. LAB can balance the gut microbiome through production of organic acids, hydrogen peroxide, lysozymes, and other metabolites which inhibit injurious toxins, as well as providing a competitive elimination mechanism at binding sites of the intestinal mucosa, thus preventing the colonization and disturbing the metabolism of pathogenic microbes [8,15,16]. LAB-based probiotics, *Lactobacillus rhamnosus* GG (LGG), are Gram-positive, rod-shaped, and non-spore forming bacteria that can produce lactic acid. They are non-toxigenic and non-pathogenic and possess potential immunostimulating activity. LGG in the human gut is considered safe and is demonstrated to have effects on innate immunity and provide clinical benefits [14,17-19]. The previous studies have covered the use of LGG as probiotics in other animals [20,21] and in fish [22].

Innate immunity, especially mucus, plays a major role in the first line of defense against infection in fish. Mucus is secreted by mucous cells or goblet cells in the epithelia of the skin, gill, stomach, and intestines of fish, where it has a lubricating property, provides mucosal protectants, and supports feeding habits [23-26]. Moreover, mucus is a viscous secretion, consisting of mucins which are glycoprotein components and a combination of other substances containing antimicrobial enzymes and possessing bacteriolytic activity, such as lysozyme, complement, C-reactive protein, lectin, and inorganic salts [16,27,28]. Mucin composition and rate of secretion vary between species and have also been observed to change in response to microbial exposure and environmental variation [29]. Mucin contents and structural changes can be detected using several special stains under various pH conditions. Nonetheless, information is still limited regarding mucous cell responses after application of probiotics.

Therefore, the aims of this study were to evaluate the effects of administration of probiotic LGG on the histopathological lesions and intestinal mucous cell responses in Nile tilapia after pathogenic *A. hydrophil* a challenge.

## Materials and Methods

### Ethical approval

This study was approved by the Kasetsart University Institutional Animal Care and Use Committee in accordance with university regulations and policies governing the care and use of laboratory animals (ACKU 02158).

### Study location and study period

These experiments were conducted at the Faculty of Veterinary Technology, Kasetsart University between October, 2018 and October 2019.

### Preparation of LAB stock: LGG

A single colony of LGG (ATCC 53103) on De Man, Rogosa and Sharpe (MRS) agar (HiMedia, India) was subcultured to MRS broth and incubated at 37°C for 24-48 h. Bacterial pellets were obtained from broth by refrigerated centrifugation at 6000 rpm and 4°C for 5-10 min. Phosphate-buffered saline (PBS) was used to wash bacterial pellets by centrifugation at 2-3 times at 6000 rpm and 4°C for 5-10 min. Following this, 20% glycerol in MRS broth was added and briefly mixed using a vortex mixer. The solution was added to individual cryovials (10 mL each), left at room temperature for 5-10 min and preserved at −20°C until needed.

### Calculation of LGG colony-forming unit (CFU) and addition to fish feed

After thawing LGG stock at room temperature, 200 mL were recovered and added into 200 mL MRS broth and incubated at 37°C for 24-48 h. Bacterial pellets were retrieved by refrigerated centrifugation at 6000 rpm and 4°C for 5-10 min, followed by centrifugation washing with PBS several times before spreading onto MRS agar plates. Each plate was then incubated at 37°C for 24-48 h before a single LGG colony was collected from each. Serial dilutions were prepared from the selected single LGG colony and plated onto another MRS agar for CFU calculation. CFU number was used to quantify the number of bacterial pellets to be added into fish feed. The fish feed was autoclaved at 121°C for 15 min and allowed to dry. LGG solution was prepared by adding PBS into the calculated number of bacterial pellets and was mixed together with fish feed inside a biological safety cabinet to provide aseptic conditions. LGG concentration in the feed was calculated as a dose of 10^10^ CFU/g feed. The LGG-mixed feed was reserved at 4°C for 3 days maximum.

### Bacterial culture and isolation: *A. hydrophila*

*A. hydrophila* originating from clinical Nile tilapia which exhibited hemorrhagic septicemia was isolated using trypticase soy agar (TSA). Bacteria were identified using a macroscopic test, Gram staining, and biochemistry testing, all kindly provided by the Department of Microbiology, Faculty of Veterinary Science, Chulalongkorn University. Bacterial stock was obtained through selection of single *A. hydrophila* colony on TSA plates, subculture to TS broth and incubation at 28-32°C for 24-48 h. Bacterial pellets were retrieved through refrigerated centrifugation at 6000 rpm and 4°C for 5-10 min. PBS was used to wash bacterial pellets using centrifugation at 2-3 times at 6000 rpm and 4°C for 5-10 min. Following this, 20% glycerol in TS broth was added and briefly mixed using a vortex mixer. The solution was added to individual cryovials (10 mL each), left at room temperature for 5-10 min, and preserved at −20°C as *A. hydrophila* stock for later use.

### Calculation of *A. hydrophila* CFU for bacterial challenge in fish

After thawing the *A. hydrophila* stock at room temperature, 200 mL were recovered and added into 200 mL TS broth and incubated at 28-32°C for 24-48 h. Bacterial pellets were retrieved by refrigerated centrifugation at 6000 rpm and 4°C for 5-10 min. After washing with PBS several times, bacterial pellets were spread onto TSA plates. The plates were then incubated at 28-32°C for 24-48 h before a single bacterial colony was collected from each. Serial dilutions were prepared from the selected single bacterial colony and plated onto another TSA plate for CFU calculation. The CFU number was used to quantify the bacterial dose to be administered in fish.

### Animal rearing

In total, 20 Nile tilapias (weight 80-110 g) were provided courtesy of the Pathumthani Aquaculture Genetics Research and Development Center. Tanks and equipment used in the experiments were all disinfected using 10% potassium permanganate solution. Fish were reared in a tank using a circulating system filter with dechlorinated water and temperature controlled to 25°C, 5-8 ppm dissolved oxygen, and pH 6.5-8.3 throughout the experiment. Fish were fed a commercial diet at 3% bodyweight twice per day and were acclimatized for 14 days before the experiment commencing. All fish were randomly divided into four groups, each of five fish, under different conditions: Group 1, control normal diet with PBS administration through intubation; Group 2, LGG-mixed feed with PBS administration through intubation; Group 3, control normal diet with *A. hydrophila* challenge through intubation; and Group 4, LGG-mixed feed with *A. hydrophila* challenge through intubation. All fish were given feed as described earlier for a period of 14 d. On day 15, fish were orally intubated with either PBS (Groups 1 and 2) or *A. hydrophila* in PBS (7.46 × 10^8^ CFU/mL/fish) (Groups 3 and 4).

### Sample collection for histopathological examination and evaluation of intestinal mucous cell responses

Fish were euthanized on day 16 using the rapid ice-cooling method. The intestines were collected into three segments as follows: Proximal (segment connected to the stomach), middle (segment between the proximal and distal segments), and distal (segment next to the anal opening). The intestinal samples were preserved in 10% neutral buffered formalin for at least 24 h, processed using a routine histopathological technique and cut into sections 4-6 mm thick before being stained with hematoxylin and eosin (HE) to determine histopathological score. Histopathological evaluation was carried out under a light microscope. Mucous cell responses were evaluated using histochemistry, with Alcian blue (AB) at pH 2.5 used for carboxylated acid mucins, AB at pH 1.0 for sulfated acid mucins, and periodic acid-Schiff-AB (PAS-AB) at pH 2.5 for mixed (acid and neutral) mucins. Fish gill and muscle tissues were used as positive and negative controls for mucin staining, respectively. The quantification of intestinal mucous cell sizes (based on diameter) and staining character of each mucin type from the entire intestine were recorded and counted under a light microscope at 400× with a high-power field. Images from stained tissue sections (three slides for each sample, with a minimum of 20 images per slide) were analyzed using the NIS-Elements Documentation Imaging Software (NIS-Elements D, version 4.00).

### Statistical analysis

Histopathological score was expressed as a p-value and intestinal mucous cell size and number in each mucin type were expressed as mean±standard deviation (SD). All data were analyzed using SPSS^®^ Statistics v.23 software (IBM^®^) and compared using analysis of variance, followed by Tukey’s honest significant difference test. Statistical significance was tested at p<0.01 to emphasize our results which would make our data more reliable and be easy to interpret data.

## Results

### Histopathological findings of intestine (HE staining)

**Figure-1:**
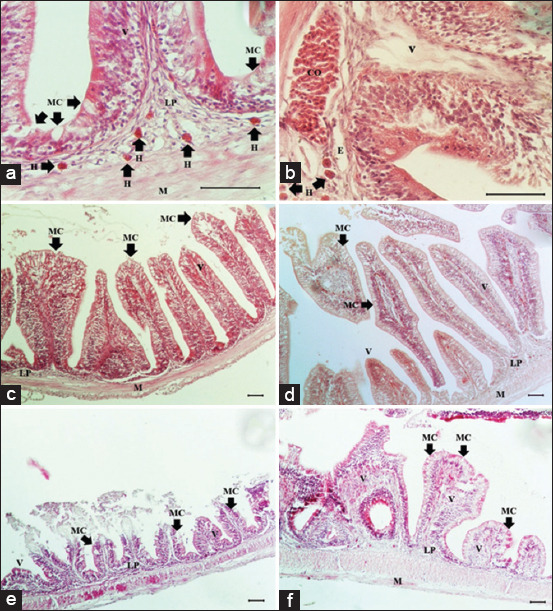
Histopathological investigation of proximal intestine of Nile tilapia with hematoxylin and eosin staining. (a) Increase number of heterophils infiltration in non-challenged fish with LGG diet; (b) lesions of *Aeromonas hydrophila*-challenged fish with normal diet; (c) characteristic of intestinal villi of non-challenged fish with normal diet; (d) characteristic of intestinal villi of non-challenged fish with LGG diet; (e) characteristic of intestinal villi of *A*. *hydrophila*-challenged fish with normal diet; (f) characteristic of intestinal villi of *A*. *hydrophila*-challenged fish with LGG diet. CO=Congestion, E=Edema, H=Heterophil, M=Muscularis, MC=Mucous cell, LP=Lamina propria, V=Villi. Bar=50 mm.

Histopathological examination found no marked pathological lesions in the entire intestines of any of the non-challenged fish. However, heterophilc infiltration in LGG-fed fish was higher without statistically significant difference compared with normal diet-fed fish (Figure-1a). Conversely, notable lesions were detected in the entire intestines of *A. hydrophila*-challenged fish, especially in the proximal part. The most common lesions observed included heterophilic infiltration, villi damage (villi shortening and sloughing off), congestion, and edema (Figure-1b), mainly observed in normal diet-fed fish. Moreover, shorter intestinal villi were observed in *A. hydrophila*-challenged fish, compared with non-challenged fish. In addition, intestinal villi had longer average length and were less damaged following *A. hydrophila* challenge in LGG-fed fish (Figure-1c-1f). Histopathological lesion scores and p-values of the three intestinal sections for fish in each group are shown in Tables-1 and 2, respectively.

**Table-1:** The average of histopathological lesion scores of proximal, middle, and distal intestine of Nile tilapia in each group (G).

Histopathological lesion types	Proximal intestine				Middle intestine				Distal intestine			
		
	G1	G2	G3	G4	G1	G2	G3	G4	G1	G2	G3	G4
Heterophils infiltration	0 (0/5)	1.2 (4/5)	1.8 (5/5)	1.6 (5/5)	0 (0/5)	0.4 (2/5)	0.8 (3/5)	0.8 (3/5)	0 (0/5)	0 (0/5)	0.4 (2/5)	0.2 (1/5)
Congestion	0 (0/5)	0 (0/5)	1.8 (5/5)	1.4 (5/5)	0 (0/5)	0 (0/5)	1.0 (3/5)	1.0 (3/5)	0 (0/5)	0 (0/5)	0.8 (3/5)	0.2 (1/5)
Intestinal villus damages	0 (0/5)	0 (0/5)	1.8 (5/5)	1.2 (5/5)	0 (0/5)	0 (0/5)	0.8 (4/5)	0.4 (2/5)	0 (0/5)	0 (0/5)	0 (0/5)	0 (0/5)
Edema	0 (0/5)	0 (0/5)	0.8 (3/5)	0.2 (1/5)	0 (0/5)	0 (0/5)	0.2 (1/5)	0.2 (1/5)	0 (0/5)	0 (0/5)	0 (0/5)	0 (0/5)

G=Group, Group 1=Fish were given the control normal diet with PBS through intubation, Group 2=Fish were given LGG-mixed feed with PBS through intubation, Group 3=Fish were given the control normal diet with *A. hydrophila* challenge through intubation, Group 4=Fish were given LGG-mixed feed with *A. hydrophila* challenge through intubation. Numbers in parentheses indicate the number of positive/number of examined fish, where 0=No histopathological change, 0.1-1.0=Mild, 1.1-2.0=Moderate, 2.1-3.0=Severe histopathological change. *A. hydrophila*=*Aeromonas hydrophila*, PBS=Phosphate-buffered saline

**Table-2:** Tukey’s honestly significant difference p-values from comparative analysis of histopathological lesion scores of P, M, and D intestine of Nile tilapia in each group.

Group		Histopathological lesion types											

		Heterophils infiltration			Congestion			Intestinal villus damage			Edema		
			
		P	M	D	P	M	D	P	M	D	P	M	D
1	2	0.015	0.768	1.000	1.000	1.000	1.000	1.000	1.000	-	1.000	1.000	-
	3	0.000**	0.251	0.314	0.000**	0.156	0.072	0.000**	0.012	-	0.072	0.752	-
	4	0.001**	0.251	0.808	0.000**	0.156	0.908	0.000**	0.314	-	0.908	0.752	-
2	1	0.015	0.768	1.000	1.000	1.000	1.000	1.000	1.000	-	1.000	1.000	-
	3	0.340	0.768	0.314	0.000**	0.156	0.072	0.000**	0.012	-	0.072	0.752	-
	4	0.662	0.768	0.808	0.000**	0.156	0.908	0.000**	0.314	-	0.908	0.752	-
3	1	0.000**	0.251	0.314	0.000**	0.156	0.072	0.000**	0.012	-	0.072	0.752	-
	2	0.340	0.768	0.314	0.000**	0.156	0.072	0.000**	0.012	-	0.072	0.752	-
	4	0.937	1.000	0.808	0.314	1.000	0.229	0.038	0.314	-	0.229	1.000	-
4	1	0.001**	0.251	0.808	0.000**	0.156	0.908	0.000**	0.314	-	0.908	0.752	-
	2	0.662	0.768	0.808	0.000**	0.156	0.908	0.000**	0.314	-	0.908	0.752	-
	3	0.937	1.000	0.808	0.314	1.000	0.229	0.038	0.314	-	0.229	1.000	-

** Differences at p<0.01 were considered significant between groups. P=Proximal, M=Middle, D=Distal

### Microscopic evaluation of intestinal mucous cell responses (histochemistry)

**Figure-2:**
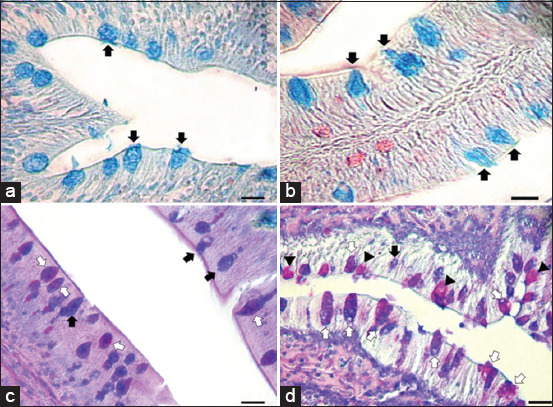
Staining characteristics of mucin in mucous cells in epithelial lining of proximal intestine using special staining. (a) Acid mucin using AB pH 2.5 (black arrow); (b) acid mucin using Alcian blue (AB) pH 1.0 (black arrow); (c) periodic acid-Schiff-AB staining of mucous cells in non-challenged fish with normal diet showed acid mucin (black arrow) and mixed mucin (white arrow); (d) periodic acid-Schiff-AB staining of mucous cells in *A*. *hydrophila*-challenged fish with LGG diet showed acid mucin (black arrow), mixed mucin (white arrow), and neutral mucins (arrowhead). Bar=125 mm.

**Figure-3:**
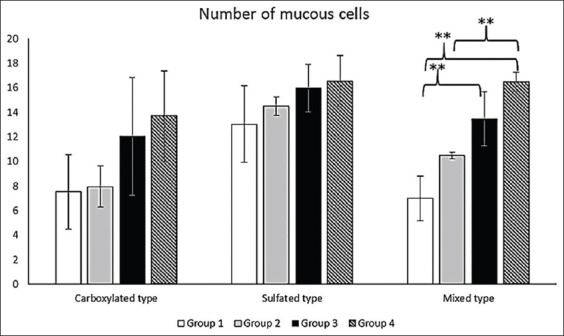
Comparison of mucin production (carboxylated, sulfated, and mixed types) from mucous cells in proximal intestine. Each bar represents the mean of four independent experiments; **=significant (p<0.01); error bars represent±standard deviation of the mean.

A comparative study was carried out on intestinal mucin production in all four groups. Positive mucins were divided into three major types: Carboxylated, sulfated, and mixed mucins. Carboxylated mucins were stained dark blue using AB at pH 2.5, while sulfated mucins were stained light blue using AB at pH 1.0. In addition, mixed mucins were stained mixed magenta-and-blue color using PAS-AB. Positive mucous cells of all types were located in the epithelial lining of intestinal surfaces. The staining characteristics of each mucin type are shown in Figure-2a-2d. No significant differences were observed regarding the sizes of any mucous cell types along the entire intestine between any groups. On the other hand, significant changes in mucous cell numbers for some mucin types were observed in some parts of the intestine. There were no significant differences in numbers of carboxylated, sulfated, or mixed mucous cells for the middle and distal intestine between any groups. Interestingly, significant (p<0.01) increase in mixed mucous cell number was observed only in the proximal intestine, which was correlated with mainly intestinal lesions within 24 h after *A. hydrophila* challenge. This significance was observed for all challenged fish, compared with non-challenged fish with a normal diet (Figure-2c and 2d). Furthermore, a significant (p<0.01) increase was also observed in LGG-fed fish with *A. hydrophila* challenge, compared with LGG-fed fish without challenge (Figure-3). Regarding the proximal intestine alone of fish from Groups 1 to 4, the mean±SD values for carboxylated mucous cell numbers were 7.53±3.01, 7.95±1.68, 12.05±4.77, and 13.70±0.78, respectively. The mean±SD values for sulfated mucous cell numbers were 13.03±3.12, 14.51±0.75, 15.97±1.92, and 16.55±2.10, respectively. The mean±SD values for mixed mucous cell numbers were 7.00±1.81, 10.49±0.27, 13.48±2.22, and 16.48±0.78, respectively. Although no significant difference was observed in mixed mucous cell numbers between normal diet-fed fish and LGG-fed fish, this cell type tended to be present in higher numbers in LGG-fed fish than in normal diet-fed fish.

## Discussion

The innate immune response is the first line of defense against invading pathogens. In fish, mucus acts as an important innate defense mechanism against pathogens at the mucosal membrane [25]. In this location, the intestinal barrier contains mucous-secreting cells which provide lubrication, support the mucosal immune response, and serve as an important form of defense [30]. Mucous-secreting cells can effectively prevent intestinal bacteria, chemicals, and endotoxins from transferring into the body [31-33].Previous studies regarding the immunomodulatory properties of probiotics in fish have mainly discussed their impacts on systemic immunity [6,8,9,12]. In contrast, few studies have so far discussed the immunomodulatory features of probiotics in mucosal surfaces with regard to changes in mucus composition [34]. In the present investigation, we aimed to examine whether LGG could prevent intestinal mucosa damage caused by pathogenic infection. To accomplish this, we performed a challenge experiment using *A. hydrophila*, evaluating histopathological lesions in the entire intestine and localization of mucin compositions methods in the different intestinal parts of LGG-fed Nile tilapia.

First, the histopathological lesions caused by acute infection with *A. hydrophila* consisted of villi damage, heterophil infiltration, edema, and congestion. These lesions were mostly detected in the proximal intestine, especially in *A. hydrophila*-challenged fish with a normal diet, imitating natural infection. Although there were no significant differences in histopathological lesions of the proximal intestine between non-challenged LGG-fed fish (Group 2) and non-challenged normal diet-fed fish (Group 1), heterophil infiltration was observed (p=0.015). Interestingly, greater infiltration was detected in LGG-fed fish (Figure-1a), resulting from the immunomodulating properties of LGG enhancing heterophil activation [34]. Heterophils play an important role against bacterial infection. Thus, LGG-fed fish may increase immune protection and decrease pathological lesions from bacterial infections. These findings also correspond to the previous studies which demonstrated that LGG stimulates immune responses through important molecules, such as SpaCBA pili and lipoteichoic acid [17,35]. Moreover, in *A. hydrophil* a-challenged fish, LGG-fed fish tended to have reduced lesion severity with no statistically significant difference, compared with normal diet-fed fish. This indicated that LGG had anti-inflammatory effects and helped to protect gut integrity and architecture against infection, in keeping with a previous report [36]. Together with the protective effects of LGG in the proximal intestine, these findings may have been due to low pH in this part affecting colonization of LGG [14]. Therefore, probiotics might pre-encounter pathogenic bacteria at mucosal-binding sites in the intestines, interfering with adhesion, and colonization capacity. In addition, probiotics have been shown to enhance protective effects through improvement of epithelial barrier function and secretion of antimicrobial peptides [17,37]. A previous report suggested that the optimal dosage of LGG for intestinal colonization was ≥5 × 10^9^ CFU/d [19]. In addition, short-term (14 d) and long-term (2 months or greater) feeding of probiotics have proven to be effective in enhancing disease resistance in tilapia through non-specific immune functions, including enhancing phagocytic ability of leukocytes, neutrophil migration, and respiratory burst [38]. In the present study, the experiment was carried out using dietary probiotic LGG at a dose of 10^10^ CFU/g twice per day for a 14 d feeding period. This might have resulted in the large localized innate immune responses together with fewer proximal intestinal lesions in LGG-fed fish.

Second, the present study examined mucous responses in the entire intestine. In this study, carboxylated, sulfated, and mixed mucous cells were distributed throughout the entire intestine, while small amounts of sulfated mucin were observed in the proximal intestine, in keeping with a previous report by Phrompanya *et al*. [26]. Interestingly, the present study showed significant changes for mixed mucous cell numbers in the proximal intestine only. This may be the result of intestinal lesions being found mostly in the proximal intestine. In the proximal intestine of non-challenged fish, LGG seemed to stimulate mucous cell proliferation of all mucin types (especially mixed mucous cells) compared with normal diet-fed fish (Figure-3). This may have been caused by the oral application of LGG, which can induce changes and interactions with carbohydrate chains of intestinal mucins to enhance the adhesion properties [14]. Many *Lactobacillus* strains show dominant binding to neutral carbohydrate chains [14]. This may have resulted in the observed changes and accumulations of both acid and neutral (mixed) mucins in the same mucous cells of the proximal intestine. Furthermore, in all *A. hydrophila*-challenged fish, increases in all mucin types (especially mixed mucous cells) were detected in both normal diet-fed and LGG-fed fish. This indicates that *A. hydrophila* also regulates changes and edits composition of mucus glycoproteins, in keeping with the previous research [39]. The adhesion manner of pathogenic bacteria was similar to that of *Lactobacillus*, using carbohydrate chains of mucin and leading the production of appropriate mucin for colonization [14]. These results implied that both LGG and *A. hydrophila* could directly affect cellular responses. In addition, a cell type shift appeared to be signaled by a combination of LGG feeding and *A. hydrophila* challenge (Figure-2c and 2d). Thus, changes in mucous cells and their composition, including mucins, are influenced by various endogenous and exogenous factors, for example, infection [23]. It has also been hypothesized that microbes (including both LGG and *A. hydrophila*) ferment sugar for growth, producing short-chain fatty acids which have direct and/or indirect effects on cell proliferation [40]. This might have contributed to the proliferation of mucous cells in LGG-fed- and *A. hydrophila*-challenged fish. A significant proliferation and increase of this cell type were observed; however, its functions are not well defined. One previous study suggested that mucous cells (mixed-mucin type) were distributed in the intestinal mucosa in other fish species, where they may serve to promote digestive functions in different environments [41]. Increased volumes of neutral mucins also had a protective effect in acidic conditions [26]. Therefore, the change in neutral and acid mucin contents in mucous cells may affect intestinal mucosal immunity against bacterial infection.

## Conclusion

LGG-fed Nile tilapia demonstrated in decreasing of pathological lesions such as maintaining villi height, less congestion, and increasing population of intraepithelial heterophils infiltration in the intestine against *A. hydrophila*-induced intestinal damage. Furthermore, the change in intestinal mucosal immunity that focused on mucin contents revealed the significant increase of the mixed mucous cell numbers in the proximal intestine. The mixed mucous cells might be one of the important mucosal immune responses in Nile tilapia after *A. hydrophila* infection. However, bacterial inhibition mechanisms, immune-related components, and other roles of mixed mucous cell needed to be further investigated.

## Authors’ Contributions

SN created the research and experimental design, performed the laboratory experiment, data analysis, and wrote the research summary. KS carried out the sample collection and helped in the laboratory work. CS carried out the data and statistical analysis. WR helped for data interpretation. All authors read and approved the final manuscript.
